# Effect of Reinforcement of Oral Health Education Message through Short Messaging Service in Mobile Phones: A Quasi-Experimental Trial

**DOI:** 10.1155/2016/7293516

**Published:** 2016-01-31

**Authors:** Harish C. Jadhav, Arun S. Dodamani, G. N. Karibasappa, Rahul G. Naik, Mahesh R. Khairnar, Manjiri A. Deshmukh, Prashanth Vishwakarma

**Affiliations:** ^1^Department of Public Health Dentistry, JMF's ACPM Dental College, Dhule, Maharashtra 424001, India; ^2^Department of Public Health Dentistry, Dr. Hedgewar Smruti Rugna Seva Mandal's Dental College, Hingoli, Maharashtra 431513, India; ^3^Department of Public Health Dentistry, Bharati Vidyapeeth Dental College and Hospital, Sangli, Maharashtra 416416, India; ^4^Department of Public Health Dentistry, Swargiya Dadasaheb Kalmegh Smruti Dental College, Nagpur, Maharashtra 441110, India

## Abstract

*Objective*. This paper aims to assess the effectiveness of reinforcement of oral health education message through short messaging service (SMS) in mobile phones.* Material and Methods*. 400 subjects from two colleges (200 from each college) belonging to 18–20 years age group possessing mobile phones were randomly selected and baseline examination of oral hygiene and gingival status was carried out using Oral Hygiene Index (OHI) and Gingival Index (GI). Oral health education was provided to all the subjects. Oral health education message was reinforced through short messaging service (SMS) in mobile phones for the subjects belonging to the intervention group. There was no such reinforcement for the control group. Follow-up examinations were done at the end of 1st, 2nd, 3rd, and 6th month. After the 3rd month, subjects of the intervention group did not receive oral health education message through short messaging service (SMS) and were followed up after next three months. Compiled data was analyzed using SPSS version 16 statistical software.* Result*. Mean OHI and GI scores in intervention group were significantly (*p* < 0.01) less than those of control group after the 2nd, 3rd, and 6th month.* Conclusion*. Reinforcement of oral health education message through short messaging service (SMS) is effective media to improve oral health.

## 1. Introduction

Many current-day health problems in individual and community are associated with lifestyle changes. Adopting unhealthy lifestyle is responsible for chronic diseases such as obesity, heart diseases, and cancer [1]. Oral diseases are no exception to this. Oral diseases are predominantly man-made attributing to his/her lifestyle [1, 2].

Human society is undergoing continuous transformation through the harnessing of information and knowledge from the various technologies that in turn have affected our value systems, power structures, everyday routines, and environment [3]. The development in technology has influenced the individual and the society. One among them is the influence of mobile on all sections of society [4]. Mobile phone usage has increased globally; over half of the world's 6.5 billion people now use mobile phone services [5]. Total wireless subscription in India stands at 952.34 million (in urban 553.45 million whereas in rural 398.89 million) at the end of January 2015 [6]. Mobile based innovations are quickly emerging as the new frontiers in transforming health due to fast-growing penetration of mobile phones into remote areas [5]. Mobile short messaging service (SMS) has high penetration and developed into a powerful, real-time communication medium [7].

The innovations and increase in usage of mobile phones have prompted for rapid communication as well as utilization for accessing personal and reliable health information [8]. Health information regarding prevention and treatment of diseases can be given to people through an affordable and cost-effective medium like short messaging service. Health education is a process of transmission of knowledge. The goal of planned health education program is not only to bring about new behavior but also to reinforce and maintain healthy behavior that will promote and improve individual, group, or community health [9]. One to one approach in oral health education is promising in improving oral hygiene, but it is time-consuming and impractical from community perspective. Available health education models have their own limitations to adopt healthy lifestyles such as slow feedback mechanism, expensive apparatus and training required limited access in rural areas, and an inability of customization for the target population [10]. Substitution of personal instruction by other means of communication has been investigated, such as the use of self-educational manuals and audio-visual aids.

People can be motivated through periodic prompts that can encourage healthy behaviors in them [11]. Reinforcement is one of the most important principles of health education which helps to adopt healthy behavior and lifestyles [12]. Text messaging is able to elicit healthier behaviors, such as adherence to treatment guidelines, smoking cessation, dietary advice, and exercise regimes that can prevent the development of certain behavior-related diseases [12, 13]. Sparse data is available regarding the use of mobile to deliver dental health across the globe and in India. Moreover, there is limited literature evidence regarding the effect of reinforcement of oral health education message through mobile phones on oral health. Hence, an attempt has been made to assess the effectiveness of reinforcement of oral health education message through short messaging service (SMS) using mobile phones among 18–20-year-old BSW (Bachelors in Social Work) students of North Maharashtra University, Jalgaon, Maharashtra, India.

## 2. Material and Methods

The present study was a quasi-experimental controlled trial conducted on 400 subjects from two different social work colleges (P. J. Nehru College of Social Work, Amalner, Jalgaon, and Dr. Babasaheb Ambedkar College of Social Work, Morane (Nakane), Tal. Dist. Dhule) in North Maharashtra region. These two colleges were randomly selected from 5 social work colleges situated in North Maharashtra region using lottery method. Both colleges were well apart from each other (almost 45 kms) so that students of both colleges could not interact with each other and they were unaware about randomization and mode of intervention in the research. Permission to conduct the research was obtained from higher authorities of both colleges. Ethical clearance was obtained from Institutional Review Board of ACPM dental college, Dhule, and informed written consent was obtained from all the participants prior to the study.

### 2.1. Inclusion Criteria

Subjects in the age group of 18–20 years possessing personal mobile phones with SMS facility and agreement to comply with the study visits were included in the study.

### 2.2. Exclusion Criteria

Subjects who did not have personal mobile phones and medically compromised subjects were excluded.

### 2.3. Study Design

Based on the data obtained from pilot study, keeping *α* at 5% (*p* < 0.05), power at 80%, considering Cohen's medium effect size 0.5, sample size for each group was determined to be 200. Since we had two groups (intervention and control), final sample size was determined to be 400.

Aim and objectives of the research were explicitly explained to both institutional higher authorities and permission to conduct the research was obtained from them. Thereafter details of the research were explained to all the students of both institutions and 200 subjects from each college satisfying the eligibility criteria were randomly selected. Baseline data using a specially prepared and pretested proforma was obtained from the 200 study subjects from each college which included demographic details. Intraoral examination was done to assess oral health using Oral Hygiene Index (given by John C. Green and Jack R. Vermillion in 1960) and Gingival Index (developed by Loe H. and Silness J. in 1963).

After the collection of baseline data, oral health education was provided to all the subjects of both colleges using common risk factor approach (oral hygiene practices, diet, habits such as smoking and alcohol use, stress, and trauma). As these causes are common to a number of other chronic diseases, adopting a collaborative approach is more rational than one that is disease specific [2]. Hence, common risk factor approach was employed in current study. Oral health education was given through PowerPoint presentation (20-minute presentation by investigator himself); demonstration of proper brushing technique on brushing model using “Modified Bass Technique” and information on use of interdental aids were also given. The benefit of using such audio-visual aids is that sound and sight can be combined together to create a better presentation in terms of better understanding on the part of the subjects. Subjects were then allowed to ask their doubts and comprehensive explanation was given to clarify their doubts.

Later intervention group and control group (college) were selected randomly by using lottery method by a person who was not known to the examiner. The examiner was blinded to the grouping. Oral health message included the information regarding proper oral hygiene practices, effects of harmful habits, and importance of proper intake of diet. The message was reinforced through short messaging service (SMS) from mobile phones for the subjects belonging to the intervention group. No other oral health information was provided to the intervention group during the study period. The messages were sent by the person who was unknown to the examiner and examiner was kept blind to the group receiving the message. Health related messages both in English and in local language (Marathi) were sent. Each of the two messages in both languages was sent to the intervention group twice a week for the period of 3 months. After the 3rd month, messages were ceased to be sent. No such oral health message or any such kind of health education was given to the participants belonging to the control group after randomization.

### 2.4. Following Oral Health Education Message Was Drafted through SMS to Be Sent to the Intervention Group

The message was as follows: “Hi, brush your teeth twice daily with toothbrush and toothpaste to avoid dental diseases. Avoid snacking in between the meals and rinse your mouth properly after every meal. Stay away from tobacco as it is the main cause of cancer, gum diseases, lung diseases and heart diseases.”

### 2.5. Testing of the SMS

After the initial framing of the oral health education message, it was sent randomly to 30 subjects to assess their opinion regarding the comprehension, relevance, and practicality of following the message. The required and relevant changes were made in the SMS. Again the procedure was repeated and the oral health education message was finalized.

### 2.6. Examination and Calibration

All the oral examinations as well as oral health education presentations were performed by a single trained and calibrated examiner. Hence, only intraexaminer reliability was determined. To determine intraexaminer reliability, the oral examination of 25 randomly selected subjects was repeated on different dates. The results so obtained were subjected to Kappa Statistics. The Kappa coefficient value for intraexaminer reliability was 0.87 which is interpreted as very good.

The examination was performed under natural light in the classroom. A set of instruments, namely, mouth mirror, explorer numbers 5 and 23, and periodontal probe, were used for each individual patient separately. Intraoral examination was done using Oral Hygiene Index and Gingival Index to collect the clinical data after the 1st, 2nd, and 3rd months from both groups. After the 3rd month, SMS to reinforce health education were ceased to be sent to the intervention group. From the 3rd month to the 6th month, no other oral health education was imparted to study participants. Then intraoral examination of both groups was done using Oral Hygiene Index and Gingival Index after six months of baseline data collection.

### 2.7. Statistical Analysis

Compiled data was analyzed using Statistical Package for Social Science (SPSS) version 16 statistical software. The intervention and control groups were compared according to age and gender using nonparametric Pearson's chi square test. Continuous data was presented as mean and standard deviation. Mean OHI and mean GI at different intervals were compared in between all subjects of intervention and control group by unpaired *t*-test. Overall changes in mean OHI and mean GI scores within intervention and control groups were compared using ANOVA test followed by post hoc analysis.

## 3. Results

### 3.1. Demographics of the Participants

Table 1 shows gender-wise distribution of study participants between the two groups. Chi square test showed no significant difference (*p* > 0.05) in the gender-wise distribution between intervention and control groups indicating a good match (Table 1).

### 3.2. Oral Hygiene Condition of Participants

Comparison of mean OHI score at different intervals between intervention and control groups showed no significant difference in mean OHI score at baseline (*p* = 0.283) and after 1st month (*p* = 0.580) in between intervention and control groups. However, mean OHI score in intervention group was significantly less than that of control group after the 2nd, 3rd, and 6th month (*p* < 0.01) (Table 2).

### 3.3. Gingival Condition of Participants

When mean GI scores between intervention and control group were compared at different intervals, it was found that there was statistically no significant difference in mean GI score at baseline (*p* = 0.394) and after 1st month (*p* = 0.849) in between intervention and control groups. But mean GI score in intervention group was less than that of control group after the 2nd, 3rd, and 6th month (*p* < 0.01) (Table 3).

### 3.4. Post Hock Comparison

The mean OHI scores (Table 4) and the mean GI scores (Table 6) of the subjects in intervention and control groups were highly significantly different (*p* < 0.001) across baseline and after 1 month, 2 months, 3 months, and 6 months.

The result of the post hoc test (with least significant difference) shows difference of mean OHI scores between each of the two time intervals to be highly significant (*p* < 0.001) in both intervention and control groups except between baseline and 3 months scores in control group (*p* = 0172) (Table 5). Similarly, difference of mean GI scores between each of the two time intervals was highly significant (*p* < 0.001) in both intervention and control groups except between 2 months and 6 months (*p* = 0.06) in the intervention group and between baseline and 3 months (*p* = 0.271) which was not statistically significant (Table 7).

## 4. Discussion

From the results, it can be seen that, in intervention group, baseline mean OHI and GI scores linearly decreased up to the 3rd month and gradually increased thereafter till the 6th month, but it was less than baseline mean OHI score and GI score. In control group, mean OHI and GI scores showed reduction after one month but thereafter linearly increased up to the 6th month. After the 6th month, mean OHI and GI scores were more than those of baseline scores.

In the present study, there was an improvement in oral health from baseline to the 1st month in both intervention and control groups which was not statistically significant (*p* = 0.58). It may be due to the fact that oral health education provided before the start of the study might have the positive effect on the oral health behavior up till one month. Hence, there was an improvement in mean OHI and GI scores of all the participants.

After baseline data collection, oral health message was reinforced through short messaging service (SMS) from mobile phones for only the subjects belonging to the intervention group, which might have kept them aware of the importance of oral health and motivated them to maintain proper oral health. Study participants in intervention group might have been more aware, concerned about their health, and more positive towards health, thus taking proper measures like proper brushing and flossing to maintain good oral health. It indicates that reinforcement at regular intervals through SMS helps to adopt healthier practices and improve oral health. These findings are in agreement with study conducted by Sharma et al. which shows that text messaging was more effective than pamphlets in improving knowledge, attitude, and practices of mothers [14]. Mean OHI and mean GI scores in intervention group were less than those of control group after the 2nd, 3rd, and 6th month. These results are in concordance with the studies conducted by Eppright et al. in orthodontic patients [15].

Limited literature is available on effect of reinforcement on oral health through SMS, but SMS reminders are effective in smoking cessation, treatment guidelines, behavior change, and so forth. The results are similar to the studies conducted by Shetty et al. showing significant improvements in the health outcomes of diabetic patients [16]. Similar results obtained in the study conducted by Koshy et al. on attendance reminders for ophthalmic patients showed that attendance rates were increased in patients who received SMS reminder compared to patients who did not receive SMS reminder [17]. Similarly Huang et al. indicated that SMS intervention enabled patients to consume their medication on time [18].

Apart from SMS intervention, other studies have been conducted using different health interventions showing positive effect on health [19, 20]. Approaches for oral health education other than personal approach have also been used which have resulted in improvement in oral health following intervention. One such study was conducted by Harnacke et al. who employed computer based training to teach either Fones technique or Modified Bass Technique. Computer presentation resulted in improvement of oral hygiene skills and gingivitis using Fones technique when compared to control groups [21]. However, no such difference was seen in participants following Modified Bass Technique. Similar results were obtained in a population based survey conducted by Gholami et al. using mass media campaign through TV channels. The study demonstrated a significant impact of the mass media campaign on Iranian adults' knowledge regarding periodontal health and disease [22]. Zotti et al. evaluated the influence of a mobile application-based approach for domestic oral hygiene maintenance in improving oral hygiene compliance and oral health in a group of orthodontic patients. The study showed positive results in improving oral hygiene compliance of adolescent patients and in improving their oral health [23].

The present study showed that maintenance of improved oral health over longer time periods requires prolonged, repeated instructions, as explained in a study conducted by Ivanovic and Lekic [24]. Apart from the reinforcement of health education through SMS to intervention group, regular visits of the investigator to collect the data, visit to dentist to seek care might have contributed to better oral health.

However, in control group, there was no improvement in oral health; probably lack of reinforcement might have resulted in poor oral health. Investigator's visit to both colleges for collecting data at every month during study period might have motivated maintaining good health behavior casting only for few days. Impact of dental examination alone might have been responsible for improvement of oral health but for limited time period. Later on the Hawthorne effect might have reduced reflecting the deterioration of oral health [25].

After cessation of intervention from the 3rd month to the 6th month in intervention group, there was an increase in mean OHI and GI scores similar to control group. Lack of oral health education through SMS within this period and lack of investigator's visits to participants might have resulted in relapse of OHI and GI scores towards baseline. Also subjects underwent their academic examinations within this period. Hence, they might have been suffering from stress due to which their oral health might have deteriorated [26]. These results are confirmatory with studies conducted by Schou showing the immediate effects on the dental health status, but these effects disappeared or decreased from the 3rd month to the 6th month after dental health education program [27].

The findings of the present study show that oral health is improved after provision of health education; reinforcing health education helped motivate maintaining good oral health. Still the study had an inherent limitation in terms of the design of the study, that is, quasi-experimental design which lacks a true randomization. Beholding the internal validity limitations of the study, generalization of the results seems to be questionable. Hence, we propose future research with the most valid study design among various population groups to assess the efficacy of this intervention.

## 5. Conclusion

SMS through mobile phone emerging as a new tool helps elicit healthier behaviors and reinforcement of oral health education message through short messaging service (SMS) using mobile phones can be effective media to improve oral health. As a Public Health Dentist, we should motivate the policy-makers to recommend telecom sector companies as a social responsibility to send free of cost oral health educational SMS at community level for taking the society to pinnacles of glory.

## Figures and Tables

**Table 1:** Gender-wise distribution between the intervention and control group.

Gender	Group		Total
	Intervention	Control	
Male	137	149	286
Female	63	51	114
Total	200	200	400
*p* *value*	*p* > 0.05		

**Table 2:** Comparison of mean OHI score at different intervals between intervention and control group.

Mean OHI score	Intervention	Control	*p* value
At baseline	3.79 ± 1.54	3.63 ± 1.39	0.283 (NS)
After 1 month	3.14 ± 1.32	3.21 ± 1.31	0.580 (NS)
After 2 months	2.64 ± 1.14	3.38 ± 1.35	<0.01
After 3 months	2.32 ± 1.07	3.56 ± 1.37	<0.01
After 6 months	2.88 ± 1.11	3.99 ± 1.46	<0.01

NS, not significant.

**Table 3:** Comparison of mean GI score at different intervals between intervention and control group.

Mean GI score	Intervention	Control	*p* value
At baseline	0.31 ± 0.32	0.29 ± 0.29	0.394 (NS)
After 1 month	0.23 ± 0.26	0.24 ± 0.27	0.849 (NS)
After 2 months	0.15 ± 0.17	0.25 ± 0.28	<0.01
After 3 months	0.10 ± 0.13	0.28 ± 0.29	<0.01
After 6 months	0.16 ± 0.16	0.35 ± 0.32	<0.01

NS, not significant.

**Table 4:** Within group comparison of mean OHI scores at each interval.

Groups	Interval	Mean	Standard deviation	*F* value	*p* value
Intervention	At baseline	3.79	1.540	109.211	<0.001
	After 1 month	3.141	1.3248		
	After 2 months	2.636	1.1441		
	After 3 months	2.319	1.0768		
	After 6 months	2.88	1.113		

Control	At baseline	3.63	1.393	108.454	<0.001
	After 1 month	3.213	1.3129		
	After 2 months	3.382	1.3529		
	After 3 months	3.561	1.3861		
	After 6 months	3.99	1.464		

**Table 5:** Pairwise comparison of mean OHI scores within intervention and control group using post hoc test.

Groups	(*I*)	(*J*)	Mean difference (*I* − *J*)	*p* value
Intervention	At baseline	After 1 month	0.651	<0.001
	At baseline	After 2 months	1.155	<0.001
	At baseline	After 3 months	1.472	<0.001
	At baseline	After 6 months	0.910	<0.001
	After 1 month	After 2 months	0.504	<0.001
	After 1 month	After 3 months	0.821	<0.001
	After 1 month	After 6 months	0.259	<0.001
	After 2 months	After 3 months	0.317	<0.001
	After 2 months	After 6 months	−0.245	<0.001
	After 3 months	After 6 months	−0.563	<0.001

Control	At baseline	After 1 month	0.420	<0.001
	At baseline	After 2 months	0.251	<0.001
	At baseline	After 3 months	0.072	0.172 (NS)
	At baseline	After 6 months	−0.357	<0.001
	After 1 month	After 2 months	−0.169	<0.001
	After 1 month	After 3 months	−0.348	<0.001
	After 1 month	After 6 months	−0.777	<0.001
	After 2 months	After 3 months	−0.180	<0.001
	After 2 months	After 6 months	−0.609	<0.001
	After 3 months	After 6 months	−0.429	<0.001

NS, not significant.

**Table 6:** Within group comparison of mean GI scores at each interval using ANOVA test.

Groups	Factor 1	Mean	Standard deviation	*F* value	*p* value
Intervention	At baseline	0.313	0.32	71.673	<0.001
	After 1 month	0.231	0.26		
	After 2 months	0.153	0.17		
	After 3 months	0.097	0.13		
	After 6 months	0.165	0.16		

Control	At baseline	0.287	0.29	71.787	<0.001
	After 1 month	0.236	0.27		
	After 2 months	0.254	0.28		
	After 3 months	0.278	0.29		
	After 6 months	0.352	0.32		

**Table 7:** Pairwise comparison of mean GI scores within intervention and control group using post hoc test.

Groups	(*I*)	(*J*)	Mean difference (*I* − *J*)	*p* value
Intervention	At baseline	After 1 month	0.082	<0.001
	At baseline	After 2 months	0.160	<0.001
	At baseline	After 3 months	0.216	<0.001
	At baseline	After 6 months	0.148	<0.001
	After 1 month	After 2 months	0.078	<0.001
	After 1 month	After 3 months	0.134	<0.001
	After 1 month	After 6 months	0.066	<0.001
	After 2 months	After 3 months	0.056	<0.001
	After 2 months	After 6 months	−0.012	0.064 (NS)
	After 3 months	After 6 months	−0.068	<0.001

Control	At baseline	After 1 month	0.052	<0.001
	At baseline	After 2 months	0.033	<0.001
	At baseline	After 3 months	0.009	0.271 (NS)
	At baseline	After 6 months	−0.064	<0.001
	After 1 month	After 2 months	−0.019	<0.001
	After 1 month	After 3 months	−0.042	<0.001
	After 1 month	After 6 months	−0.116	<0.001
	After 2 months	After 3 months	−0.024	<0.001
	After 2 months	After 6 months	−0.097	<0.001
	After 3 months	After 6 months	−0.074	<0.001

NS, not significant.
